# Differences in the Interleukin Profiles in Inattentive ADHD Prepubertal Children Are Probably Related to Conduct Disorder Comorbidity

**DOI:** 10.3390/biomedicines12081818

**Published:** 2024-08-09

**Authors:** Raquel González-Villén, María Luisa Fernández-López, Ana Checa-Ros, Pilar Tortosa-Pinto, Raquel Aguado-Rivas, Laura Garre-Morata, Darío Acuña-Castroviejo, Antonio Molina-Carballo

**Affiliations:** 1 Unit of Pediatric Neurology and Neurodevelopment, Clínico San Cecilio University Hospital, Andalusian Health Service, 18016 Granada, Spain; raquelgonzavi@gmail.com (R.G.-V.); pilartortosa@yahoo.es (P.T.-P.); raquel.aguado.sspa@juntadeandalucia.es (R.A.-R.); 2 Department of Pediatrics, School of Medicine, University of Granada, 18071 Granada, Spain; 3 Biohealth Research Institute Granada (ibs. GRANADA), University Hospital of Granada, 18016 Granada, Spain; laura.garre.sspa@juntadeandalucia.es (L.G.-M.); dacuna@ugr.es (D.A.-C.); 4 Research Group on Cardiorenal and Metabolic Diseases, Department of Medicine and Surgery, Faculty of Health Sciences, Cardenal Herrera-CEU University, CEU Universities, 46115 Valencia, Spain; acheca1987@gmail.com

**Keywords:** ADHD, prepubertal children, attention deficit, cytokines, methylphenidate, oppositional defiant conduct disorder

## Abstract

Inflammatory cytokines are involved in attention deficit hyperactivity disorder (ADHD), a highly prevalent neurodevelopmental disorder. To quantify the baseline levels of pro- and anti-inflammatory cytokines and their changes after methylphenidate (MPH), a total of 31 prepubertal children with ADHD were recruited and subclassified into only two ADHD presentations—ADHD attention deficit (*n* = 13) or ADHD combined (*n* = 18). The children were also screened for oppositional defiant conduct disorder (ODCD) and anxiety disorder. Blood samples were drawn at 09:00 and after 4.63 ± 1.87 months of treatment. Four pro-inflammatory cytokines (interleukin-1beta (IL-1β), IL-5, IL-6, tumor necrosis factor-alpha (TNF-α)) and three anti-inflammatory cytokines (IL-4, IL-10, IL-13) were measured using a Luminex^®^ assay. For statistics, a factorial analysis was performed in Stata 15.1. Overall, there were no statistically significant differences in the interleukin (IL) values induced by treatment. When grouped by presentation, the differences were present almost exclusively in ADHD-AD, usually with a profile opposite to that observed in ADHD-C, and with interactions between comorbid factors, with IL-1β (*p* = 0.01) and IL-13 (*p* = 0.006) being the ones reaching the greatest statistical significance. These differences are probably related to the ODCD factor, and they disappear after treatment. In conclusion, the changes observed in cytokine levels in prepubertal children only in the ADHD-AD presentation are probably related to comorbidities (specifically ODCD) and are mitigated after treatment.

## 1. Introduction

ADHD is a highly prevalent neurodevelopmental disorder in children characterized by inattention and/or hyperactivity–impulsivity [1], which alters psychological, social, academic, and occupational functions [2]. The current global ADHD prevalence in children and adolescents is 7.2%, persisting in up to 3% of adults [2,3]. At the beginning of school age, ADHD largely predominates in males (3:1), with hyperactivity–impulsivity being the most common presentation (previously subtype). Neurodevelopment transforms symptoms towards an inattentive presentation [4], with a reduction in the highest incidence seen in men [5].

ADHD is a clinical diagnosis, with the onset of symptoms occurring before the age of 12 years [6], and is manifested in multiple settings [7]. The disorder is associated with multiple comorbidities [8] and different physical conditions [9]. Oppositional defiant conduct disorder (ODCD) represents the most frequent comorbidity, as it is present in more than two-thirds of patients. In addition, ADHD increases the risk of psychosis in adulthood [10].

ADHD is a multifactorial disorder, in which multiple genes and neurobiological factors are influenced by social and environmental risk factors (educational styles, family interaction, or the presence of parental psychopathology) [11]. It is estimated that up to 75% of ADHD cases are due to genetic inheritance [12], and the remaining 25–35% are attributable to acquired factors.

During brain development, stress [13], infections, and immune activation promote neuroinflammation with the progression of neurodevelopmental disorders, in part activating the kynurenin pathway of tryptophan metabolism [14] and leading to increased levels of nitric oxide, IFN-γ, and other inflammatory substances, such as cytokines [15,16]. Furthermore, chronic inflammation is related to the pathogenesis and progression of several psychiatric and mental health disorders [17,18].

Cytokines, a large group of signaling molecules, play an important role in neurodevelopmental processes, affecting glial cell development and neural and synaptic maturation [19]. Interleukins, a subfamily of cytokines, are secreted proteins that regulate the inflammatory process, which is defined by the balance between pro-inflammatory and anti-inflammatory cytokines. Pro-inflammatory cytokines, such as the interleukins (IL) IL-1β [20], IL-5 [21], IL-6 [22], and tumor necrosis factor alpha (TNF-α), mediate early responses and amplify inflammatory reactions, whereas anti-inflammatory cytokines, including IL-4, IL-10 [23], and IL-13 [24], have the opposite effect, as they limit inflammatory reactions.

Subclinical neuroinflammation has been demonstrated in ADHD [25] in both children [26] and adults [27], with increased oxidative stress demonstrated in both animals [28] and pediatric patients [29]. An association has been detected between elevated levels of pro-inflammatory cytokines and the severity of symptoms in children with ADHD [30]. Specifically, IL-6 has been proposed to be a hallmark of ADHD pathogenesis, since an increase in IL-6 [31] may alter attention and memory through its effects on synaptic plasticity [32].

Due to the temporal stability of inattention across pediatric ages compared to other ADHD symptoms (ADHD-HI) [4], it is necessary to delve deeper into the neurobiological underpinnings of inattention. Based on this knowledge, we hypothesized that the profile of a group of ILs usually measured in a clinical setting would be involved in ADHD neurobiology or the response to methylphenidate (MPH). Consequently, our study aimed to examine several ILs in prepubertal children with ADHD and to evaluate the direction of such putative changes after chronic treatment with MPH.

## 2. Materials and Methods

### 2.1. Subjects

**Figure 1:**
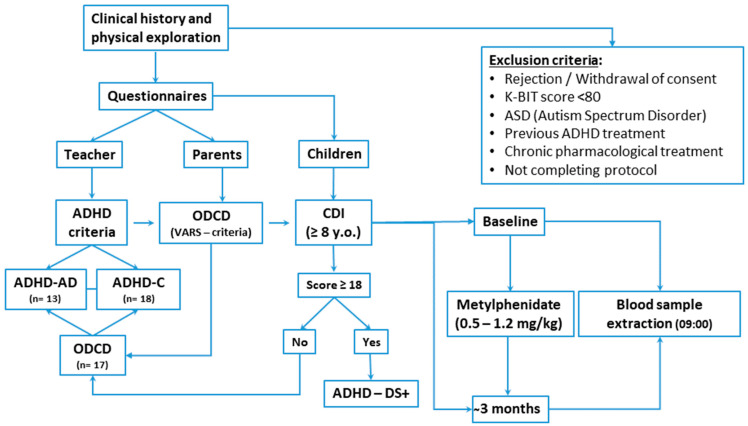
Study design scheme and timeline.

Children (*n* = 31; 23 males, 8 females) aged 6–10 years (7.64 ± 1.33) were included in a prospective, quasi-experimental, and open-label clinical trial. The hospital-based sample consisted of patients with ADHD who were assessed at least twice; consequently, every patient acted as their own control. All included children met the DSM-5-TR ADHD criteria [6]. The study design scheme and timeline are included in Figure 1.

Written informed consent was obtained from all parents and responsible individuals, since they were all under 12 years of age. The study design was approved by the Hospital Research Ethics Committee and it was conducted in accordance with the 2013 Revision of the Declaration of Helsinki. The exclusion criteria included the following: (1) a K-BIT score <80; (2) previous or current treatment with ADHD or antiseizure drugs; (3) other neuropsychiatric, metabolic, or endocrine disorders or chronic diseases able to justify the current symptoms; and (4) refusal to participate or revocation of consent.

### 2.2. Assessment

A personal medical history, semi-structured clinical interview, and physical examination were performed. Subsequently, the following questionnaires were supplied to the parents for completion and quantitative assessment in the next clinical visit: (a) the NICHQ Vanderbilt Assessment Scales, which include the DSM-V Criteria for Deficit of Attention and Hyperactivity Scales completed by teachers and separately by parents, are used to help in the diagnostic process of ADHD in children between the ages of 6 and 12, and the teacher-rated version can be used in parallel with the parent’s version; (b) the Vanderbilt Scales for oppositional defiant conduct disorder (ODCD); (c) the Spence Children’s Anxiety Scale (SCAS) self-reporting instrument, to which children respond; (d) the Children’s Depression Inventory (CDI) [33], a screening instrument used to locate children with highly depressive symptoms, completed by patients ≥8 years of age. Based on the NICHQ Vanderbilt scores, the ADHD group was quantitatively subclassified into two clinical subgroups: (a) the children who had attention deficit (AD) if their scores were >11 for deficit of attention and <10 for hyperactivity–impulsivity, and (b) the children with combined ADHD presentation if they had scores <11 for AD and >11 for HI (hyperactivity) or scores >11 for both AD and HI.

The oppositional defiant disorder screening (items 19 to 28 of the Vanderbilt Scales) was completed by the parents. The children needed to be above the clinical cut-off score of 2 or 3 (or more) for 4 out of the 8 behaviors on questions 19–26 (total score sum ≥8 points considered pathological) and have a score 1 or 2 on any of the performance questions 36–43. Seventeen of the thirty-one children (54.83%) who were included met the criteria for the diagnosis of ODCD.

Depressive symptoms (DS) were assessed through interviews conducted with patients and their parents as respondents and quantitatively through a self-report assessment of depression for children. The CDI test comprises two subscales containing items that are more related to depression than to anxiety. We considered the sum of both subscales, with a cut-off >17 point considered pathological, to define our subgroups.

The Spence Children’s Anxiety Scale (SCAS), a self-report instrument to which children respond, consists of 44 items that measure separation anxiety, obsessive–compulsive disorder, panic, agoraphobia, social phobia, generalized anxiety, and fear of physical harm. It also contains 6 filler items that are not graded because it is intended to reduce the impact of the negative bias produced by the list of problems. The higher the score, the more severe the anxiety symptoms presented; a total score of ≥60 was used as the cut-off when interpreting the SCAS as an indicator of elevated anxiety symptoms.

All children were initially evaluated using the Kaufman Brief Intelligence Test, an abbreviated intelligence test containing a vocabulary subtest (verbal intelligence) and a matrix subtest (nonverbal intelligence).

### 2.3. Treatment

The only drug used was prolonged-release methylphenidate, initially at 0.5 mg/kg/day. The dosage was adjusted based on the response and tolerance to treatment. A dose increase was made approximately every 2 weeks until the recommended dosage was reached or due to insufficient therapeutic effect; occasionally, a lower dose was indicated when side effects were not well tolerated. The mean initial dose of MPH was 15.53 ± 5.74 mg, and the final dose was 32.43 ± 8.43 mg. At the time of recruitment, all patients were MPH-naïve, and no other pharmacological or psychological treatment was administered before the conclusion of the protocol. In addition to the full assessments included in the study, the researchers involved in the fieldwork contacted the parents by phone every 2 weeks to ensure the proper completion of the protocol.

The specific dosage escalation regime for each patient is provided in Supplementary Materials (Table S3).

### 2.4. Measurements

Fasting blood samples were taken in the absence of an acute or severe illness at 09:00. The same study protocol was repeated after 4.63 ± 1.86 months of daily MPH administration. All samples were stored at −80 °C until analyses were performed.

### 2.5. Analytical Method

ProcartaPlex multiplex immunoassays use Luminex xMAP (multi-analyte profiling) technology to enable the simultaneous detection and quantitation of up to 80 protein targets in a single 25–50 µL sample of plasma. The Luminex technology combines the efficiencies of multiplexing with the accuracy, sensitivity, reproducibility, and simplicity of an ELISA. In our study, we used the Human Cytokine 7-Plex ProcartaPlex Panel 1C, which allows the study of the immune response by analyzing seven protein targets in a single well using Luminex xMAP technology (Affymetricx, LabClinics, Málaga, Spain).

### 2.6. Statistics

Each group of variables was tabulated, distinguishing between the possibility of them being quantitative or qualitative variables and whether or not they followed a normal or binomial distribution. The descriptive data are presented as means and standard deviations (SDs). For comparative analyses, all data were subjected to normality testing with the Kolmogorov–Smirnov test, and Bartlett’s test was used for equal variances. For each study variable, within-patient and between-patient comparisons were carried out according to the following factorial model: comparisons between (a) groups of patients with two categories, namely PAD and PHI; comparisons between (b) patients nested in the ADHD presentation groups and ODCD group; and (c) intra-patient comparison in two moments, before and after treatment. The moments represented a crossed factor with subgroups and ODCD. The subgroups and moments were fixed-effect factors, and patients represented a random-effects factor. All data were subjected to an analysis involving segregation by sex. Missing values (~15% in our sample) were substituted by Stata Multiple Imputation Data software (Version 15). For both types of comparisons (within-participants and between-participants), an analysis of variance (ANOVA: one-way and multilevel one-way) table was built and higher interactions were determined. If these were significant, multiple pairwise comparisons were made using Bonferroni’s post hoc analysis, and if not, these corrections were applied to the principal effects in the table. In all cases, the interactions were studied for levels <0.15, and the latest comparisons were considered significant at *p* < 0.05 after applying the penalty provided by the correction. When analyzing the variances in different groups, homogeneous transformations were carried out on the data using natural logarithms to achieve uniformity. All analyses were performed using STATA 15.1 software. Comparative analyses were plotted via the freely available JASP (version 0.19.0.0) software.

The anonymized patient database containing the raw values of the different ILs and their logarithmic transformation is provided as part of the supplementary files, together with all the ANOVA statistical calculations performed on STATA 15.1.

## 3. Results

The demographic and clinical characteristics of our sample, including the ADHD, ODCD, Spence, and CDI scores at baseline and after treatment are shown in Table 1. The comparisons plotted as Bayesian-repeated measures ANOVA are shown in Supplementary Figure S1. According to parental reports (NICHQ Vanderbilt scores), ADHD symptoms significantly remitted in 73.3% of participants after treatment with MPH, together with the scores referred to ODCD, depression (CDI), and anxiety (SCAS) symptoms.

The quantitative data of the aforementioned demographic and clinical characteristics before and after treatment, expressed as median and interquartile range (IQR), with 95% confidence intervals (CI), are shown in Supplementary Materials (Table S1).

### 3.1. Pro-Inflammatory Cytokines

#### 3.1.1. Interleukin-1beta (IL-1beta)

We found a statistically significant difference (F (1.46) = 5.39, *p* = 0.025) based on sex, with a considerably higher concentration (and larger dispersion of data) of IL-1 beta in boys (2.49 ± 3.9 pg/mL) than in girls (0.22 ± 0.15 pg/mL).

Based on treatment, no significant difference was observed in IL-1beta concentrations (Table 2 and the ANOVA Supplementary Materials).

**Figure 2:**
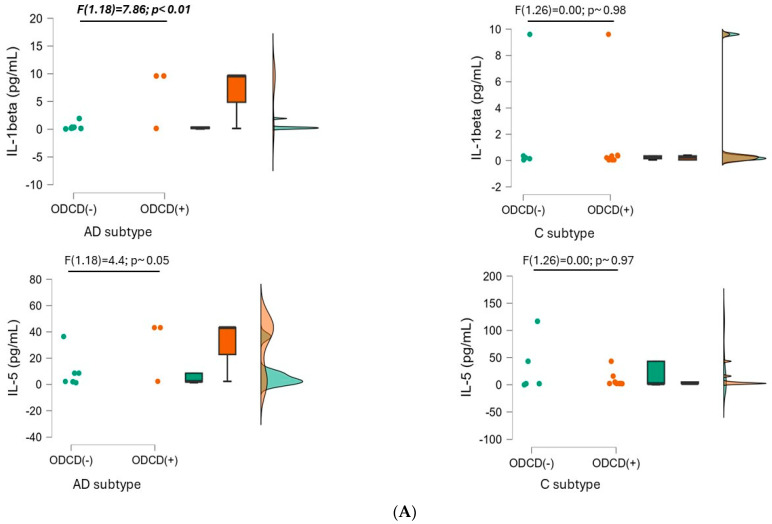

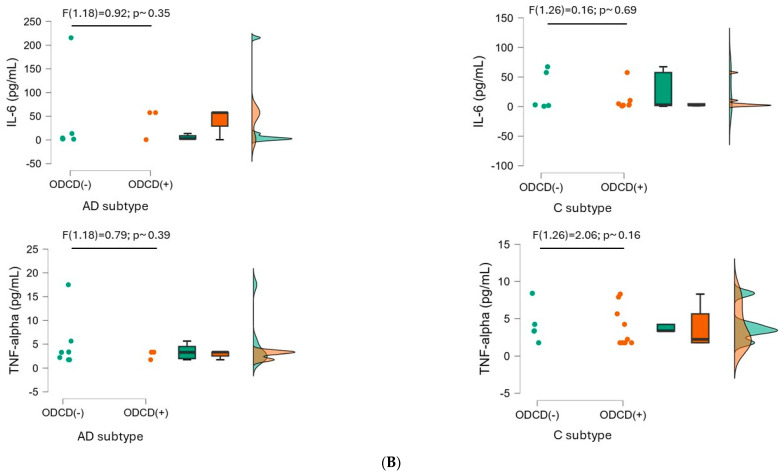
Between-patient comparison of the four pro-inflammatory cytokines. Baseline interleukin values are compared between patients without oppositional defiant conduct disorder (ODCD-; in green) and patients with it (ODCD+; in orange) for each ADHD subtype: inattentive (AD) and combined (C). Data are reported as individual datapoints, as box plots (where the black bold line represents the median; hinges show the 25th and 75th percentiles; whiskers represent the 1.5 interquartile ranges beyond the hinges), and as data density estimates following the Gaussian kernel method. (**A**) Comparisons inf IL-1beta and IL-5; (**B**) comparisons in IL-6 and TNF-alpha.

When comparing both ADHD presentations (ADHD-AD and ADHD-C), no statistically significant differences in IL-1beta levels were found. Nonetheless, within the AD subtype, concentrations significantly increased in those with comorbid ODCD (6.46 ± 4.86 pg/mL; [F (1.18) = 7.86, *p* < 0.01]). For the ADHD-C subtype, however, no significant differences were observed based on the presence of ODCD, although those without this comorbidity tended to show higher levels of IL-1beta (Figure 2A).

The factorial analysis revealed statistical significance (F (5.32) = 3.15, *p* < 0.02) due to the form of presentation (F = 7.26, *p* < 0.01), with interactions between the three factors (F (5.32) = 6.1, *p* < 0.006) (ANOVA Supplementary Materials).

#### 3.1.2. Interleukin-5 (IL-5)

The large data dispersion hindered the finding of significant sex-related differences in IL-5 concentrations.

Similarly, no significant differences in IL-5 levels were observed after treatment with MPH (post-treatment: 12.80 ± 20.28 pg/mL vs. baseline: 16.36 ± 26.78 pg/mL; F(1.46) = 0.27, *p*~ 0.6) (Table 2 and ANOVA Supplementary Materials).

Baseline concentrations of IL-5 did not significantly differ between the two ADHD presentations (ADHD-AD and ADHD-C); neither did they for the presence of ODCD within each ADHD subtype. However, IL-5 levels were likely to increase in the presence of ODCD in the AD subtype (29.52 ± 21.20 vs. 10.78 ± 20.64 pg/mL in the absence of ODCD; F(1.18) = 4.4, *p*~0.05), whereas the opposite seemed to occur in the ADHD-C presentation (without ODCD: 21.69 ± 37.57 vs. 8.62 ± 13.14 pg/mL in patients with ODCD) (Figure 2A).

A significant difference was observed in the factorial analysis for IL-5, due to higher concentrations of this interleukin in the ADHD-AD subtype (F(2.11) = 5.9; *p* < 0.02) (ANOVA Supplementary Materials).

#### 3.1.3. Interleukin-6 (IL-6)

In relation to sex differences, a larger concentration of IL-6 was found in boys (28.26 ± 58.66 vs. 2.45 ± 1.31 pg/mL in girls; F(1.46) = 3.54; *p*~0.07).

As for the aforementioned cytokines, no statistically significant differences in IL-6 levels were observed after treatment (Table 2 and ANOVA Supplementary Materials).

However, IL-6 concentrations were doubled in patients with the ADHD-AD subtype in comparison with those with the combined presentation (36.56 ± 77.06 vs. 13.11 ± 22.24 pg/mL). Focusing on each ADHD subtype, the presence of ODCD did not seem to significantly change IL-6 concentrations, probably due to the large data dispersion (39.01 ± 91.24 vs. 30.84 ± 29.45 in ADHD-AD without and with ODCD, respectively; 19.51 ± 28.67 vs. 9.55 ± 17.69 in ADHD-C without and with ODCD, respectively) (see Figure 2B). The factorial analysis including all factors indicated a statistically significant higher concentration of IL-6 for the ADHD-AD subtype (F(2.11) = 5.62; *p*~0.02) (ANOVA Supplementary Materials).

#### 3.1.4. Tumor Necrosis Factor-Alpha (TNF-α)

No significant differences attributed to sex were observed for TNF-α concentrations.

Post-treatment TNF-α levels were nearly twice the baseline concentrations (Table 2), although no statistical significance was reached. (4.18 ± 3.55 pg/m, baseline; 7.29 ± 8.89 pg/mL, post-treatment) (Table 2, ANOVA Supplementary Materials).

Significant differences in TNF-α levels were neither observed between the two ADHD presentations (5.7 ± 7.85 pg/mL in ADHD-AD vs. 5.76 ± 6.24 pg/mL in ADHD-C), nor in relation to the presence of ODCD within each ADHD subtype. As seen in Figure 2B, the tendency of TNF-α concentrations showed by patients with AD subtype and ODCD was the opposite to the ones with ODCD in the ADHD-C presentation (4.66 ± 5.69 vs. 8.13 ± 11.84 pg/mL, without and with ODCD in ADHD-AD, respectively; 8.39 ± 9.47 vs. 4.30 ± 2.82 pg/mL in ADHD-C without and with ODCD, respectively).

Statistical significance (F(2.11) = 4.08; *p* < 0.047) was found during the factorial analysis, due to the anxiety factor, but only in the ADHD-AD subtype (ANOVA Supplementary Materials).

Descriptive statistics of the pro-inflammatory interleukins shown as median (IQR) and 95% CI based on ODCD for each ADHD subtype are included in Supplementary Table S2.

### 3.2. Anti-Inflammatory Cytokines

#### 3.2.1. Interleukin-4 (IL-4)

There were no statistically significant differences in IL-4 concentration related to sex.

Neither were they observed after treatment with methylphenidate (Table 2 and the ANOVA Supplementary Materials).

**Figure 3:**
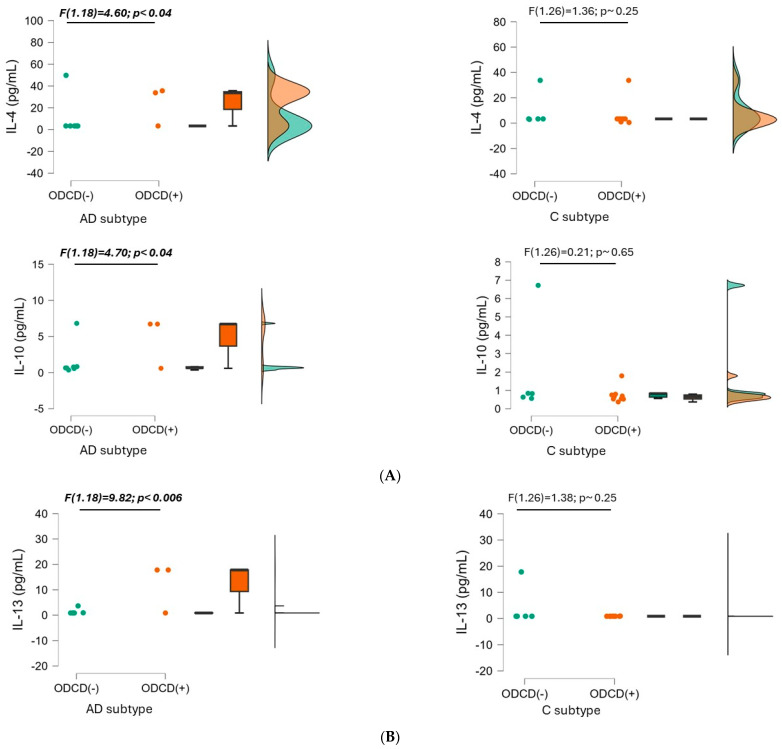
Between-patient comparison of the three anti-inflammatory cytokines. Baseline interleukin values are compared between patients without oppositional defiant conduct disorder (ODCD-; in green) and patients with it (ODCD+; in orange) for each ADHD subtype: inattentive (AD) and combined (C). Data are reported as individual datapoints, as box plots (where the black bold line represents the median; hinges show the 25th and 75th percentiles; whiskers represent the 1.5 interquartile ranges beyond the hinges), and as data density estimates following the Gaussian kernel method. (**A**) Comparisons in IL-4 and IL-10; (**B**) comparisons in IL-13.

IL-4 concentrations were non-significantly higher in the ADHD-AD (16.1 ± 23.66 mg/mL vs. 7.26 ± 10.66 pg/mL in ADHD-C; F (1.46) = 2.3, *p*~0.14). However, significant differences were found in IL-4 levels in relation to ODCD symptoms within the AD presentation (24.1 ± 16.1 pg/mL in patients with ODCD vs. 12.7 ± 26.01 pg/mL in patients without ODCD; F(1.18) = 4.6, *p* < 0.046). The profile shown by patients with ADHD-C seemed to be the opposite (higher IL-4 levels in the absence of ODCD), without reaching statistical significance (see Figure 3A).

When including the set of factors analyzed, a significant increase in IL-4 was revealed in patients with ADHD-AD (F (2.11) = 10.3, *p* < 0.003), due to the influence of the ODCD factor (ANOVA Supplementary Materials).

#### 3.2.2. Interleukin-10 (IL-10)

IL-10 concentrations in boys nearly doubled those in girls (2.09 ± 2.58 vs. 0.9 ± 0.38 pg/mL, respectively), but without reaching statistical significance.

No statistically significant differences were observed after treatment (Table 2 and the ANOVA Supplementary Materials).

Although significance was not reached for the differences when comparing the two ADHD subtypes (2.37 ± 2.83 pg/mL in ADHD-AD vs. 1.47 ± 1.9 pg/mL in ADHD-C), IL-10 concentration was significantly higher in patients with ADHD-AD and ODCD (3.93 ± 3.06 in the presence of ODCD, 1.70 ± 2.56 pg/mL in the absence of ODCD; [F (1.18) = 4.7, *p* < 0.04]). The inverse trend, however, was observed for the ADHD-C (1.21 ± 1.44 pg/mL with ODCD vs. 1.93 ± 2.55 pg/mL without ODCD) (Figure 3A).

Interestingly, the presence of anxiety symptoms seemed to influence IL-10 concentrations. In the absence of anxiety, IL-10 levels were practically identical in both presentations (1.74 ± 2.34 pg/mL) and were considerably higher in the presence of anxiety, especially in the ADHD-AD presentation (4.1 ± 3.71 pg/mL). However, data interpretation was limited by the small sample size and the large dispersion of the data.

The model that included the three factors was near statistical significance (F (5.32) = 2.32, *p*~0.07)), with differences due to the ODCD factor; the interaction between the three factors was significant (F (5.32) = 4.85, *p* < 0.015). In the factorial analysis segregated by presentation, the concentration of IL-10 was closely linked to the presence of ODCD symptoms, but only in patients with ADHD-AD (F (2.11) = 5.8, *p* < 0.02) (ANOVA Supplementary Materials).

#### 3.2.3. Interleukin-13 (IL-13)

A non-significantly higher concentration of IL-13 was found in boys.

Treatment with methylphenidate did not appear to significantly change IL-13 concentrations either (Table 2 and ANOVA Supplementary Materials).

Distinguishing between ADHD subtypes, the baseline concentrations of IL-13 in ADHD-AD (4.54 ± 7.04 pg/mL) were twice the levels found in ADHD-C (2.09 ± 4.52 pg/mL). This difference disappeared after treatment, due to a decrease in IL-13 levels in ADHD-AD patients, together with an increase in the ADHD-C presentation (Table 2). The differences found did not reach statistical significance, probably due to the large data dispersion.

The presence of ODCD induced significant differences in the ADHD-AD subtype (9.47 ± 9.13 pg/mL in the presence of ODCD vs. 2.54 ± 4.76 pg/mL in the absence of ODCD; F (1.18) = 9.82, *p* < 0.006). An inverse profile was shown by patients with the ADHD-C presentation, without statistical significance (4.35 ± 7.09 pg/mL without ODCD vs. 3.81 ± 6.41 pg/mL with ODCD; F (1.26) = 1.37, *p*~0.25). These differences are illustrated in Figure 3B).

Descriptive statistics of anti-inflammatory interleukins shown as median (IQR) and 95% CI based on ODCD for each ADHD subtype are included in Supplementary Table S2.

## 4. Discussion

To date, this is the first study reporting putative changes induced by treatment with MPH in the interleukin profiles in children with ADHD. In addition, this is the first study segregating the analyses by ADHD presentation and by ODCD as an ADHD comorbidity.

In our study, we analyzed the profiles of seven cytokines in 31 pre-pubertal patients newly diagnosed with ADHD before and after 3 months of treatment with methylphenidate. In the whole group of patients, there were no statistically significant differences in response to treatment in the concentrations of the ILs analyzed. When analyzing the data grouped by the two main presentations of ADHD, we observed higher concentrations of all cytokines in those participants with ADHD-AD with comorbid ODCD, except for IL-6 and TNF-α. No differences induced by MPH were found in patients with ADHD-C with ODCD. The differences detected, including those induced by methylphenidate, were counterbalanced in the entire sample by the interactions between factors (i.e., the different concentrations and modifications of one factor depended on the level of the other factor). These changes were probably secondary to the ODCD factor to a much greater extent than being a reflection of the core symptoms of ADHD, with IL-1β and IL-13 being the cytokines that reached the greatest statistical significance.

Among the neurobiological correlates of ADHD, inflammation may play a crucial role in the pathogenesis of this disorder. ADHD may result from an exaggerated inflammatory response of the fetal central nervous system to maternal inflammation. In addition, it has been shown that inflammatory responses associated with infection during the first post-natal month are associated with the risk of ADHD at 10 years of age [32]. This finding is supported by genome-wide association studies reporting that mutations in different genes encoding proteins involved in the inflammatory response are associated with an increased risk of ADHD [34,35]. Furthermore, patients with ADHD and their mothers have a higher risk of developing chronic diseases with an immunological component [36,37].

In comparison with controls, patients with ADHD have elevated concentrations of pro-inflammatory cytokines (such as IL-6 and TNF-α) and reduced levels of anti-inflammatory cytokines (IL-4, IL-2, and IFN-γ) and brain-derived neurotrophic factor (BDNF) [31,32,38]. Furthermore, an association has been detected between elevated levels of pro-inflammatory cytokines and the severity of symptoms in children with ADHD [30,39], an association that is not found in adult patients with ADHD [40].

IL-6 and TNF-α may activate the hypothalamus–pituitary–adrenal (HPA) axis, and, in turn, glucocorticoids strongly interact with the immune system. A population-based sample of adults showed a lower increase in cortisol in subjects with elevated plasma levels of IL-6 and TNF-α, suggesting an inverse relationship between cortisol secretion and certain plasma cytokines [40].

IL-4 and IL-13 are members of the T-helper (Th)2 family of cytokines, with overlapping functions, antagonizing the Th1-driven pro-inflammatory immune response. They downregulate the synthesis of many pro-inflammatory cytokines, including IL-1, IL-6, IL-12, TNF-α, and the macrophage migration inhibitory factor (MIF) [41].

IL-10 is the cytokine with the greatest anti-inflammatory capacity, as it inhibits the production of Th1-cells and intervene in the differentiation of some cell types [42].

A meta-analysis published in 2021 found that young people aged 4–17 years with ADHD have significantly lower levels of TNF-α and unchanged levels of IL-1β, IL-6, and IL-10 in the peripheral blood [43]. This sample included a wide age-ranged group and did not consider the influence on puberty. Additionally, it also did not differentiate between presentations and comorbidities of ADHD and did not indicate whether patients had received previous psycho-pedagogic or pharmacological treatments. Furthermore, the heterogeneity of the studies included should be taken into consideration when interpreting these results.

With similar limitations, a more recent meta-analysis based on a relatively low number of studies found subclinical immune-inflammatory alterations (increase in IL-6 and decrease in TNF-α), with significant differences in the absence of pharmacological treatment in children or adolescents and without differences in adults with ADHD. Unlike the authors measuring TNF-α levels, the studies determining IL-6 levels were highly heterogeneous [44]. Neurodevelopmental processes modify the symptoms of ADHD presentation, and in adulthood the symptoms persist in up to half of the diagnosed cases [45]. Therefore, it is expected that there will be differences in immune-inflammatory factors between children and adolescents, or even adults, with ADHD.

In another recent study, a small group of twenty adolescents (five women) with ADHD showed higher concentrations of IL-1, IL-6, and TNF-α, and an overall greater pro-inflammatory profile than a healthy control group comparable in age and sex. This paper concluded that activation of the inflammatory response system is detected in adolescents with ADHD in both sexes, but slightly more pronounced in male adolescents [46].

These results support the hypothesis of an activation of the kynurenine pathway of tryptophan metabolism in ADHD [47], which may be more intense in the presence of comorbidities and less susceptible to modification by MPH treatment. This activation may be associated with higher levels of IL-6, regardless of the presence of ODCD comorbidity [32]. A subgroup of adults with ADHD could also have abnormal cytokine levels [40].

In a group of patients of both sexes aged between 6 and 14 years, MPH treatment induced improvements in the redox status, the inflammatory profile, and in response to the HPA axis [29]. In addition, MPH treatment was associated with modifications in other aspects of tryptophan metabolism, whereby (a) the indole tryptophan metabolites showed changes related to the presence of depressive symptoms [48], and (b) serotonin and melatonin showed subtle favorable changes in both serum concentrations and daily fluctuations [49]. In this sense, a “neutralization” effect by psychostimulant medications had been previously postulated in both children [50] and adults [51].

In addition to the lack of a control group, the main limitation of our study was the small sample size. No validation strategies were applied. This was a contribution based on routine care and the daily clinical practice at the hospital; the patients included may show more severe symptoms. On the other hand, the strengths of our study were the inclusion of newly diagnosed patients (who had not previously taken any pharmacological treatment) who served as their own controls and the recruitment of pre-pubertal children without other pathologies except for a small number of patients showing anxiety or depressive symptoms after a thorough assessment of the most frequent ADHD comorbidities.

## 5. Conclusions

Our data suggest that the quantification of several cytokines merits further evaluation as a useful objective marker of ADHD-AD subtype/presentation together with the clinical diagnosis and may be also useful for the evaluation of pharmacotherapy in school children with the ADHD-AD presentation and comorbid ODCD. We need well-designed clinical trials and studies to confirm the potential role of neuroinflammation in ADHD.

## Figures and Tables

**Table 1:** Demographics characteristics of the sample, ADHD presentations, and CDI and SCAS scores for the ADHD group before and after treatment (within-patient comparisons).

	Baseline ADHD	Post-Treatment ADHD	Statistics	
	(*n* = 31)	(*n* = 31)	t	*p*
Age (years)	7.65 ± 1.33	8.1 ± 1.5	1.24	0.21
Height (cm)	130.31 ± 9.49	133.50 ± 8.03	1.43	0.16
Weight (kg)	33.03 ± 8.08	35.9 ± 7.77	1.16	0.15
Body Mass Index (kg/m^2^)	19.28 ± 4.42	19.90 ± 3.34	0.62	0.54
Attention Deficit (ADHD-AD)	17.48 ± 4.42	5.38 ± 4.25	10.99	0.00001 ***
Combined ADHD (ADHD-C)	13.19 ± 8.92	3.59 ± 4.29	5.4	0.00001 ***
OD Conduct Disorder (ODCD)	10.55 ± 7.24	4.25 ± 6.16	3.69	0.0005 ***
CDI total score (CDI)	10.2 ± 5.63	7.69 ± 3.05	2.18	0.03 *
Anxiety total score (SCAS)	30.42 ± 15.49	16.93 ± 13.15	3.7	0.0005 ***
KBIT total score	101.23 ± 11.4			

Data are expressed as means ± SDs (standard deviations). Attention deficit and hyperactive–impulsive ADHD and oppositional defiant conduct disorder: total scores for each Vanderbilt subscale. CDI (Childhood Depression Inventory): total score. SCAS Spence Scale Anxiety: total score. KBIT: Kaufman Brief Intelligence Test. * *p* < 0.05, *** *p* < 0.001.

**Table 2:** Serum interleukin values before and after methylphenidate treatment.

Interleukins		Inattentive ADHD (*n* = 13)		Combined ADHD (*n* = 18)	
		Baseline	Post-MPH	Baseline	Post-MPH
Pro-inflammatory	IL-1beta	2.26 ± 3.91	2.83 ± 4.17	1.55 ± 3.41	1.72 ± 3.35
	IL-5	14.96 ±18.21	17.83 ± 26.32	17.36 ± 32.21	9.21 ± 14.65
	IL-6	35.75 ± 67.13	37.38 ± 89.61	15.41 ± 24.85	10.81 ± 19.96
	TNF-alpha	4.39 ± 4.75	7.00 ± 10.19	4.03 ± 2.55	7.49 ± 8.24
Anti-inflammatory	IL-4	14.25 ± 18.04	17.95 ± 29.13	7.29 ± 11.25	7.23± 10.48
	IL-10	2.49 ± 2.95	2.27 ± 2.87	1.16 ± 1.63	1.77 ± 2.15
	IL-13	4.54 ± 7.04	2.89 ± 5.29	2.08 ± 4.52	3.49 ± 6.09

Values are expressed as means ± standard deviations. MPH, methylphenidate.

## Data Availability

The database and statistical results resulting from this study is available in Open Science Framework. The link will be provided by the corresponding author upon request.

## Supplementary Materials

The following supporting information can be downloaded at: https://www.mdpi.com/article/10.3390/biomedicines12081818/s1, Table S1. Demographic characteristics of the sample, ADHD presentations, and CDI and SCAS scores for the ADHD group before and after treatment, expressed as median (interquartile range) with 95% confidence intervals; Table S2. Comparisons of inteleukin (IL) levels for each ADHD subtype based on the presence of oppositional defiant conduct disorder (ODCD); Table S3. Methylphenidate dose escalation; Figure S1. Within—patient changes in demographic and clinical characteristics following treatment with methylphenidate.
