# Supplementary material for: Differences in the Interleukin Profiles in Inattentive ADHD Prepubertal Children Are Probably Related to Conduct Disorder Comorbidity
Source: Biomedicines. 2024 Aug 9;12(8):1818. doi: 10.3390/biomedicines12081818 (PMC11351999; doi:10.3390/biomedicines12081818)
Supplement: Supplementary file 1 [file biomedicines-12-01818-s001.zip › biomedicines-3085275-supplementary.pdf]

Supplementary Files

Supplementary Table S1. Demographic characteristics of the sample, ADHD presentations, and CDI and SCAS scores for the ADHD group before and after treatment, expressed as median (interquartile range) with 95% confidence intervals.

	Baseline ADHD<br>(n= 31)<br>*Median (IQR)*<br>*95% CI*	Post-treatment ADHD<br>(n= 31)<br>*Median (IQR)*<br>*95% CI*
Age (years)	7 (2.0)<br>95%CI [7.16, 8.13]	8 (3.0)<br>95%CI [7.96, 9.01]
Height (cm)	131 (14.5)<br>95%CI [126.83, 133.79]	136 (6.5)<br>95%CI [131.11, 141.12]
Weight (kg)	31.4 (12.95)<br>95%CI [30.06, 35.99]	36 (11.80)<br>95%CI [32.58, 39.89]
Body Mass Index (kg/m^2)	18.75 (5.33)<br>95%CI [18.03, 20.53]	20.08 (4.02)<br>95%CI [18.12, 21.28]
Attention Deficit (ADHD-AD)	16 (6.0)<br>95%CI [15.86, 19.10]	6 (2.75)<br>95%CI [3.94, 7.20]
Combined ADHD (ADHD-C)	30 (19.50)<br>95%CI [26.54, 34.62]	10 (8.0)<br>95%CI [6.38, 12.05]
OD Conduct Disorder (ODCD)	12 (10.5)<br>95% CI [7.69, 13.42]	0 (10)<br>95% CI [0.97, 7.53]
CDI_total score (CDI)	8.5 (9.5)<br>95% CI [7.44, 12.18]	7 (5.25)<br>95% CI [6.06, 9.31]
Anxiety total score (SCAS)	29.5 (24.25)<br>95% CI [23.88, 39.60]	16 (19.5)<br>95% [9.65, 24.22]

Attention deficit and hyperactive–impulsive ADHD and oppositional defiant conduct disorder: total scores for each Vanderbilt subscale. CDI (Childhood Depression Inventory): total score. SCAS Spence Scale Anxiety: total score. IQR=interquartile range; 95% CI=95% confidence interval.

Supplementary Table S2. Comparisons of inteleukin (IL) levels for each ADHD subtype based on the presence of oppositional defiant conduct disorder (ODCD).

Supplementary Table S2A. Predominantly inattentive ADHD subtype (AD).

	AD subtype_IL1beta		AD subtype_IL5		AD subtype_IL 6		AD subtype_TNF alpha		AD subtype_IL4		AD subtype_IL10		AD subtype_IL13	
	**ODCD-**	**ODCD+**	**ODCD-**	**ODCD+**	**ODCD-**	**ODCD+**	**ODCD-**	**ODCD+**	**ODCD-**	**ODCD+**	**ODCD-**	**ODCD+**	**ODCD-**	**ODCD+**
Median	0.255	9.600	2.224	43.200	2.550	57.600	3.285	3.325	3.340	33.752	0.667	6.720	0.870	17.800
95% CI Upper	1.073	20.011	20.382	88.215	108.472	120.368	10.296	5.048	26.193	69.233	3.692	13.448	2.251	36.438
95% CI Lower	-0.137	-7.116	-2.961	-29.075	-39.419	-43.179	-0.141	0.559	-6.253	-20.771	-0.625	-4.082	0.306	-12.125
IQR	0.195	4.728	6.537	20.445	6.723	28.508	2.516	0.783	0.000	16.131	0.194	3.055	0.034	8.465

Supplementary Table S2B. Combined ADHD subtype (C).

	C subtype_IL1beta		C subtype_IL5		C subtype_IL6		C subtype_TNF alpha		C subtype_IL4		C subtype_IL10		C subtype_IL13	
	**ODCD-**	**ODCD+**	**ODCD-**	**ODCD+**	**ODCD-**	**ODCD+**	**ODCD-**	**ODCD+**	**ODCD-**	**ODCD+**	**ODCD-**	**ODCD+**	**ODCD-**	**ODCD+**
Median	0.220	0.255	1.991	2.310	2.963	2.550	3.365	2.221	3.340	3.340	0.829	0.654	0.870	0.870
95% CI Upper	7.300	3.668	95.315	19.267	67.599	23.533	7.332	6.030	26.288	14.149	5.255	1.063	13.657	0.898
95% CI Lower	-3.151	-1.144	-29.689	-1.719	-15.441	-4.550	1.106	1.828	-7.595	-1.861	-1.417	0.423	-5.145	0.862
IQR	0.214	0.305	41.209	2.798	55.752	2.727	0.910	3.888	0.000	0.000	0.205	0.220	0.000	0.000

ODCD- = without oppositional defiant conduct disorder; ODCD+ = with oppositional defiant conduct disorder; IL1beta=IL-1 beta; TNF alpha=tumor necrosis factor alpha; 95% CI= 95% confidence interval; IQR= interquartile range.

Supplementary Table S3. Methylphenidate dose escalation

Age (years)	Weight (Kg)	Initial dose (mg)	Escalating dose (mg)	Final dose (mg)
8	43.0	18	27	36
7	39.8	18	36	54
7	32.5	18	27	36
9	30.0	18	27	36
6	28.0	10	20	30
7	27.0	10	20	30
7	28.5	18	27	27
8	42.5	18	27	36
9	49.3	27	36	54
7	28.0	18	27	36
9	34.0	10	20	30
9	25.0	10	20	30
7	40.0	20	30	40
7	23.6	18	27	27
8	39.9	18	27	36
6	38.2	10	20	30
7	24.7	10	20	27
9	42.0	10	20	30
7	24.4	18	27	27
6	21.5	10	20	30
10	45.2	18	27	27
6	24.4	18	27	27
7	32.0	18	27	36
8	27.4	18	27	36
6	27.0	18	27	36

Supplementary Files

9	42.0	10	20	30
6	31.4	10	20	30
9	31.4	10	20	30
10	48.0	18	18	18
10	30.2	10	20	30
6	23.0	10	20	20

Supplementary Files

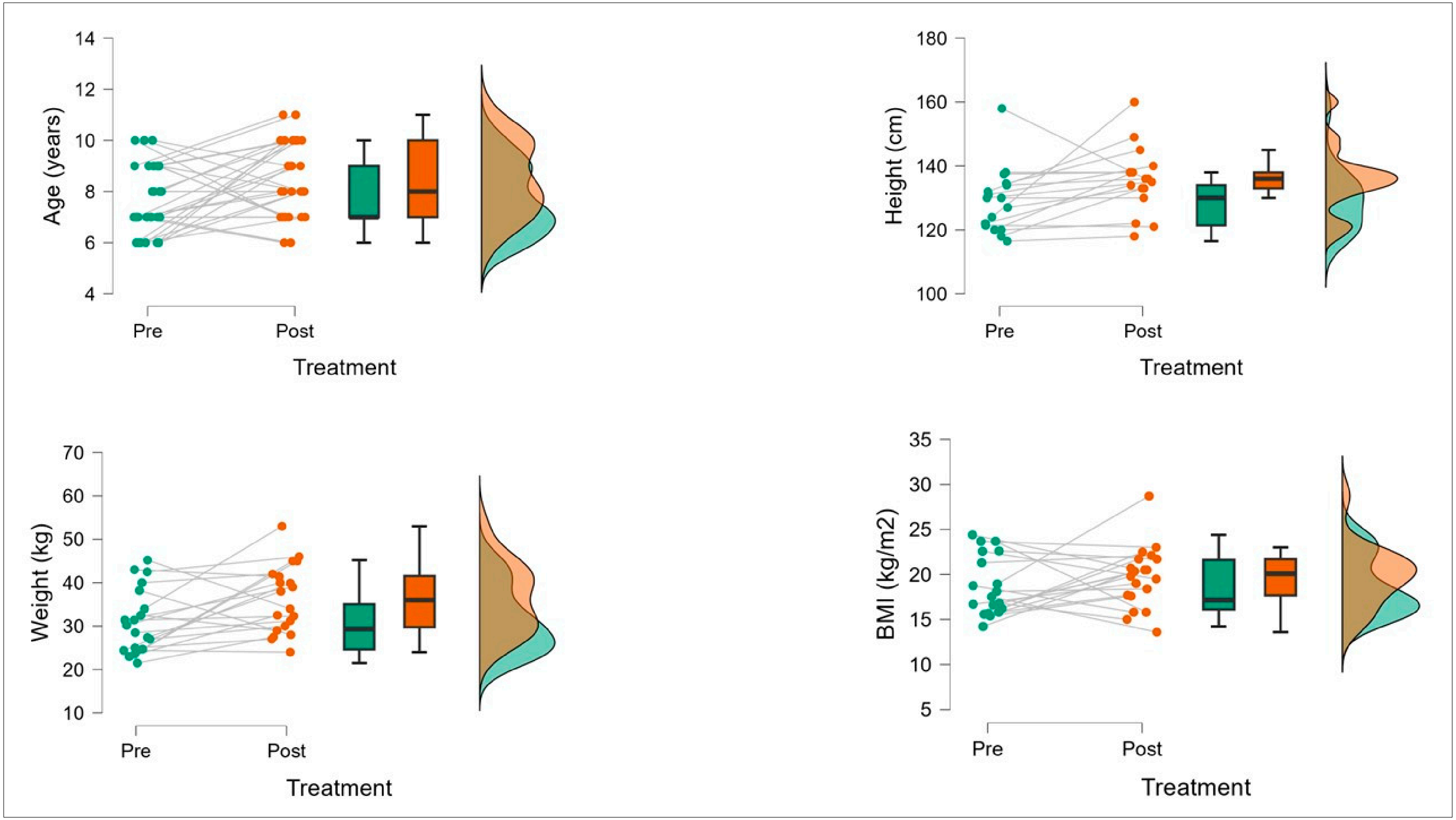

Supplementary Figure S1A.

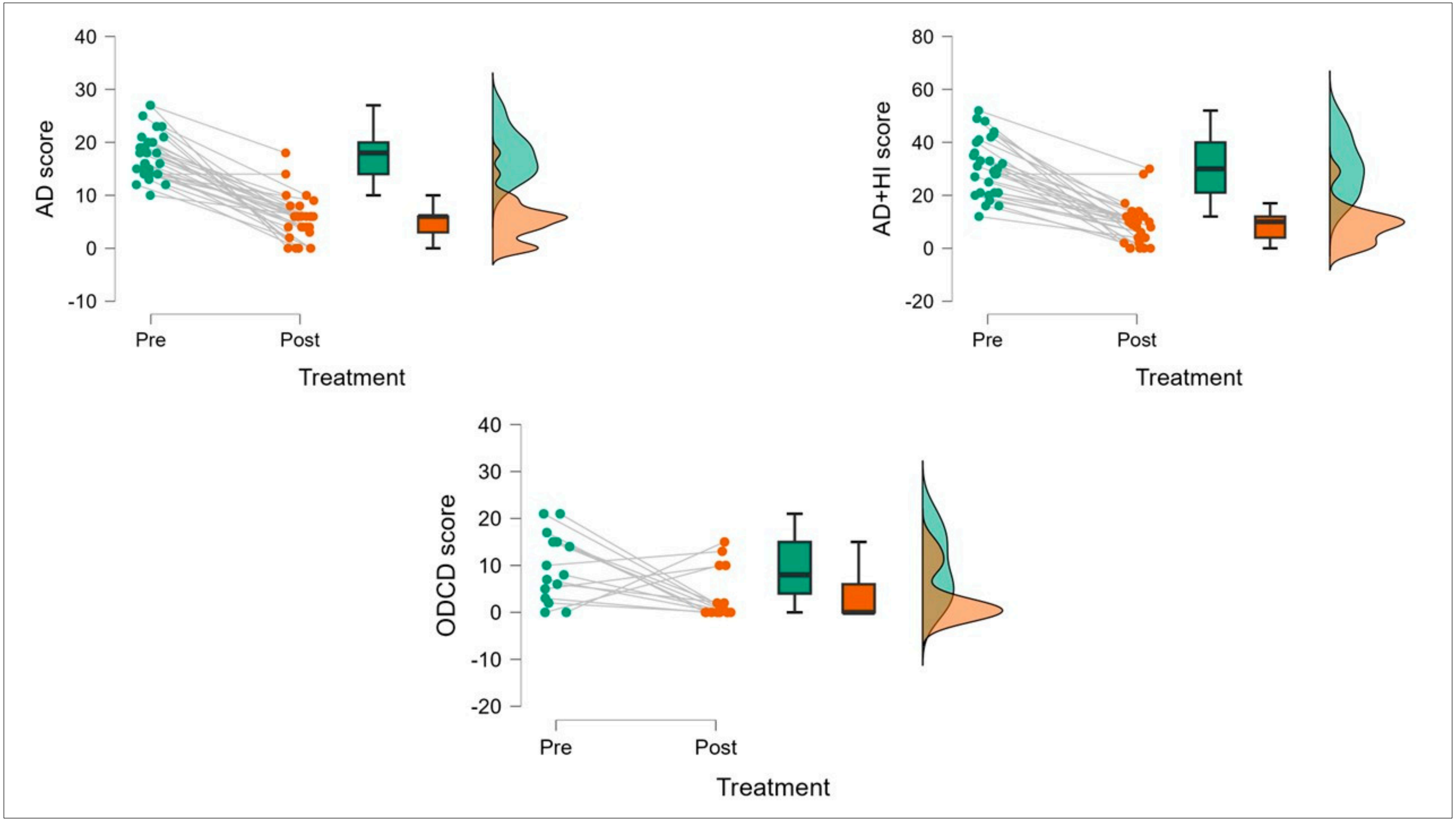

Supplementary Figure S1B.

Supplementary Files

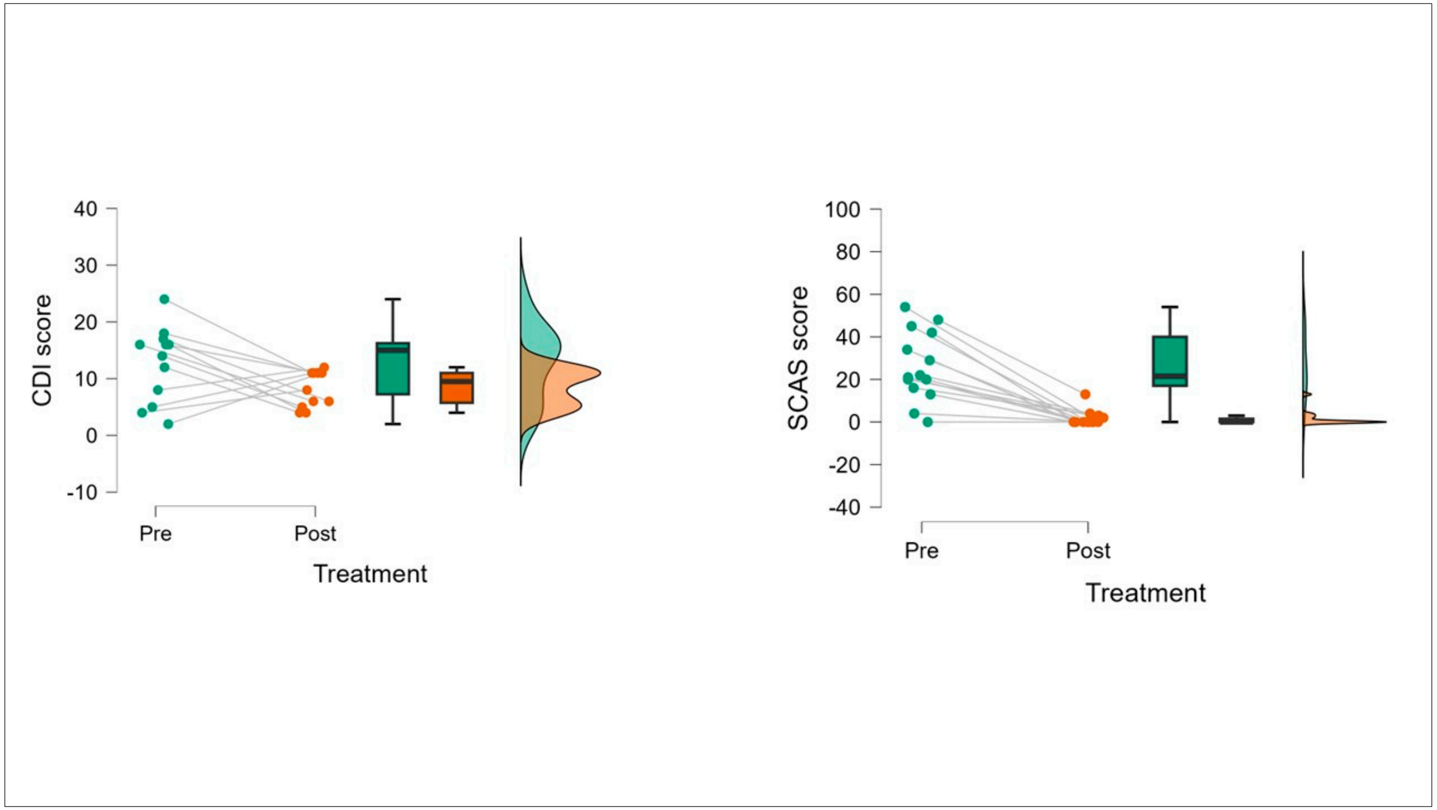

Supplementary Figure S1C.

Supplementary Figure S1. **Within—patient changes in demographic and clinical characteristics following treatment with methylphenidate**. Pre (baseline; in green) and post (after treatment; in orange) data are reported as individual datapoints, as box plots (where the black bold line represents the median; hinges show the 25th and 75th percentiles; whiskers represent the 1.5 interquartile ranges beyond the hinges) and as data density estimates following the Gaussian kernel method. . **1.A**-Changes in ge (years), height (cm), weight (kg), BMI=body mass index (kg/cm^2). **1.B-** Changes in total scores for each Vanderbilt subscale (AD=attentional deficit; AD+HI=combined; ODCD=oppositional defiant conduct disorder). **1.C-**Changes in CDI (Childhood Depression Inventory) score and SCAS (Spence Scale Anxiety) score.
